# Nitrogen-fixing *Rhizobium*-legume symbiosis: are polyploidy and host peptide-governed symbiont differentiation general principles of endosymbiosis?

**DOI:** 10.3389/fmicb.2014.00326

**Published:** 2014-06-30

**Authors:** Gergely Maróti, Éva Kondorosi

**Affiliations:** Institute of Biochemistry, Biological Research Center, Hungarian Academy of SciencesSzeged, Hungary

**Keywords:** *Rhizobium*-legume symbiosis, bacteroid differentiation, host effector molecules, plant peptides, polyploidy, endosymbiont

## Abstract

The symbiosis between rhizobia soil bacteria and legumes is facultative and initiated by nitrogen starvation of the host plant. Exchange of signal molecules between the partners leads to the formation of root nodules where bacteria are converted to nitrogen-fixing bacteroids. In this mutualistic symbiosis, the bacteria provide nitrogen sources for plant growth in return for photosynthates from the host. Depending on the host plant the symbiotic fate of bacteria can either be reversible or irreversible. In *Medicago* plants the bacteria undergo a host-directed multistep differentiation process culminating in the formation of elongated and branched polyploid bacteria with definitive loss of cell division ability. The plant factors are nodule-specific symbiotic peptides. About 500 of them are cysteine-rich NCR peptides produced in the infected plant cells. NCRs are targeted to the endosymbionts and the concerted action of different sets of peptides governs different stages of endosymbiont maturation. This review focuses on symbiotic plant cell development and terminal bacteroid differentiation and demonstrates the crucial roles of symbiotic peptides by showing an example of multi-target mechanism exerted by one of these symbiotic peptides.

## HOST-SPECIFIC INTERACTION BETWEEN THE *Rhizobium* AND PLANT PARTNERS

The bacteria which form nitrogen-fixing symbiosis with legume plants belonging to diverse groups of α- and β-proteobacteria are collectively called rhizobia (Chen et al., 2003; MacLean et al., 2007). Many α-proteobacteria are engaged in long-term interactions with higher eukaryotes. These interactions range from surface colonization through facultative symbiotic relationships to obligate intracellular pathogen or endosymbiont lifestyles. The symbiotic genes required for nodule formation, host cell infection and nitrogen fixation have been acquired by lateral gene transfer which is the primary source of genetic diversity of rhizobia. Therefore, rhizobia could be more closely related to pathogens (such as *Agrobacterium* or *Brucella*) than to each other. Rhizobia tend to have large genomes (up to 10.5 Mbp) which in fast growing rhizobia are dispersed on multiple replicons (MacLean et al., 2007). For example, *Sinorhizobium meliloti*, the endosymbiont of *Medicago* species, has a tri-parted genome; a 3.65 Mbp chromosome and two megaplasmids, pSymA and pSymB (1.35 and 1.68 Mbp) both of which are indispensable and carry the majority of symbiotic genes. However, many *S. meliloti* strains contain further auxiliary medium sized plasmids and thus, the *S. meliloti* genome may contain up to 9,000 genes (Barnett et al., 2001; Capela et al., 2001). In contrast to rhizobia, obligate endosymbionts of insects usually possess a strongly reduced (160–450 Kbp) genome which ensures their multiplication and codes for a few specific biosynthetic pathways including those satisfying the host’s need (Moran et al., 2008; Price et al., 2011). These incredibly reduced genomes are nevertheless amplified compensating the diminished genome with a polyploid DNA content.

The plant partners of rhizobia belong to the *Leguminosae/Fabaceae* family. Nitrogen fixing symbiosis has evolved in several lineages, but not all legumes form symbiosis. Hitherto 12,000 nodulated legume species are known and each has its own *Rhizobium* partner(s). The symbiosis is triggered by nitrogen starvation of the host plant which has to select its *Rhizobium* partner from billions of bacteria in the rhizosphere. This is achieved by secretion of flavonoid signal molecules from the root which act as chemo-attractants but most importantly as inducers of the *Rhizobium* nodulation genes (Oldroyd, 2013). These genes are required for the production of bacterial signal molecules; the Nod factors (NFs) which trigger the nodule developmental program in the host plant (Walker and Downie, 2000). The NFs are lipochitooligosaccharide molecules that carry host specific substitutions on the terminal sugar residues and characteristic lipid chains, which are recognized by LysM-type host receptors and are required both for nodule development and bacterial infection. Interestingly, the ancient symbiosis of land plants with arbuscular mycorrhizal (AM) fungi operates with similar lipochitooligosaccharide signal molecules, the Myc factors which are perceived by similar but different LysM-type receptors as the NFs (Abdel-Lateif et al., 2012; Oldroyd, 2013). The Myc factors and NFs activate a common signaling pathway but after the involvement of the common symbiotic genes conserved in plants, the pathways deviate; one leading to nodulation, the other for AM symbiosis.

Plant infection and nodule formation are intricate processes; Nod factors play distinct roles in nodule organogenesis and root hair infection. Moreover, beside Nod factors, various bacterial surface polysaccharides are crucial for efficient infection (Fraysse et al., 2003). In most legumes, the rhizobia enter the host via the root hairs where by invagination of the plasma membrane an infection thread (IT) is formed that contains the multiplying bacteria and grows towards the root cortex. A less frequent and ancient mode of infection occurs via cracks on the root surface of certain legumes.

## DETERMINATE AND INDETERMINATE NODULE DEVELOPMENT

Nodule development requires mitotic reactivation of cortical cells leading to nodule primordium formation which then differentiates into nitrogen-fixing root nodules providing microaerobic condition in the central zone for functioning of the oxygen sensitive nitrogenase enzyme in the bacteroids. Depending on the transient and persistent nature of host cell proliferation, the nodules can be either determinate or indeterminate type (Terpolilli et al., 2012; Kondorosi et al., 2013). Determinate nodules have no meristem and contain homogenous population of symbiotic cells. Determinate nodules develop for example on *Phaseolus vulgaris* and *Lotus japonicus* roots.

**FIGURE 1:**
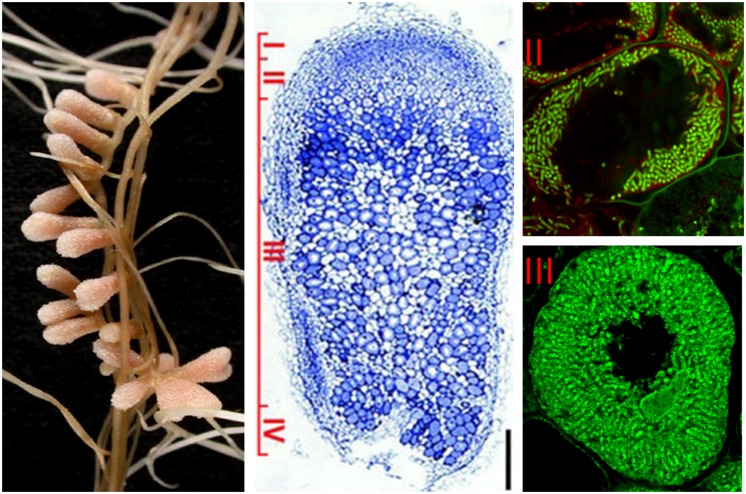
**Structure of nitrogen-fixing root nodules formed in *S. meliloti* –* M. truncatula* symbiosis.** The different nodules zones are indicated on the longitudinal nodule section: (I) meristem, (II) infection zone, (III) nitrogen fixation zone, (IV) senescence zone. Symbiotic cells in zone II contain the differentiating endosymbionts while in zone III the host cytoplasm is fully packed with long nitrogen-fixing bacteroids. Endosymbionts stained with Syto9 have green fluorescence.

To the contrary, the active cell division is maintained in the indeterminate nodules. A nodule meristem is present in the apical region (zone I) which by constant generation of new cells provokes continuous growth and elongated nodule shape. The cells leaving the meristem do not divide anymore and enter a differentiation phase. The infection thread releases the bacteria into the submeristematic cells, which differentiate gradually along the 12–15 cell layers of the infection zone (zone II), leading to the development of nitrogen-fixing symbiotic cells in nodule zone III (**Figure 1**; Franssen et al., 1992). *Medicago sativa, M. truncatula, Vicia sativa*, and *Pisum sativum* are examples of plants forming indeterminate nodules.

## GROWTH OF SYMBIOTIC CELLS INVOLVES AMPLIFICATION OF THE HOST GENOME BY ENDOREDUPLICATION CYCLES

Extreme plant cell enlargement can be observed in both the determinate and indeterminate nodules. The cytoplasm of a nitrogen-fixing symbiotic cell hosts about 50,000 bacteroids. To accommodate such a high number of endosymbionts, the host cells grow. In *M. truncatula* nodules the volume of the nitrogen-fixing cells is 80-fold larger than that of the diploid meristematic cells. The growth of infected cells occurs stepwise in zone II and is the consequence of repeated endoreduplication (ER) of the genome without mitosis. In zone II the cell cycle machinery is still active but the lack of mitotic cyclins inhibits mitosis and transforms the mitotic cycles to endoreduplication cycles (Cebolla et al., 1999). This is achieved by the cell cycle switch CCS52A protein that by the destruction of the mitotic cyclins induces repeated rounds of genome duplication leading to the formation of gradually growing polyploid cells (Roudier et al., 2003; Kondorosi and Kondorosi, 2004). In *Medicago* species the ploidy levels can reach 64C representing 64-fold higher DNA content compared to the haploid cells (C corresponds to the haploid DNA content; Vinardell et al., 2003). Down-regulation of CCS52A in *M. truncatula* had no effect on primordium formation but was detrimental for nodule differentiation indicating that the ER cycles and formation of large highly polyploid cells are essential for nodule functioning (Vinardell et al., 2003). Interestingly, cortical cells containing AM fungi are also polyploid, as well as the nematode-feeding giant root cells (Favery et al., 2002; Genre et al., 2008). Similarly, insect symbiotic cells, the bacteriocytes harboring intracellular endosymbionts are also large and polyploid (Nakabachi et al., 2010). In angiosperm plants, polyploidy is frequent and the specific inherited pattern of polyploidy in different organs, tissues and cell types suggest that it could be a major source of the specialized physiology of host cells (Nagl, 1976; Edgar et al., 2014). Beside cell growth, the multiple gene copies, lack of chromosome condensation can contribute to higher transcriptional and metabolic activities. However, association of polyploidy with different cell functions suggests an impact of polyploidy also on the architecture of nucleosomes and on the epigenome controlling activation or repression of specific genomic regions. Accordingly, the polyploid genome content of symbiotic cells appears to be a prerequisite for nodule differentiation and for the expression of most symbiotic host genes (Maunoury et al., 2010).

### DIFFERENT FATES OF NITROGEN FIXING BACTEROIDS

The bacteria released from the IT are present in the host cytoplasm as organelle-like structures, called symbiosomes. The bacteria have no direct contact with cytoplasm as they are surrounded by a peribacteroid membrane, known also as symbiosome membrane (SM). The bacteroid, the SM and the space between them comprise the symbiosome (Catalano et al., 2004). The SM during its formation reflects its plasma membrane origin, later modifications of its composition open new, specialized roles at the host-endosymbiont interface (Limpens et al., 2009; Ivanov et al., 2012; Brear et al., 2013; Sinharoy et al., 2013). The bacteroids multiply in the growing host nodule cells to a certain cell density, adapt to the endosymbiotic life-style and microaerobic conditions and mature to nitrogen-fixing bacteroids. The form and physiology of bacteroids can be, however, strikingly different in the various legumes. In certain legume hosts, the nitrogen-fixing bacteroids have the same morphology as cultured cells; this type of bacteroids can revert to the free-living form. In other associations, the bacteroids are irreversibly transformed to polyploid, enlarged, non-cultivable endosymbionts. These terminally differentiated bacteroids can be elongated and even branched and 5- to 10-fold longer than the free-living cells or can be spherical from 8 to at least 20-fold amplified genome depending on the host (Mergaert et al., 2006; Nakabachi et al., 2010). Terminal differentiation of bacteroids is host controlled, evolved in multiple branches of the *Leguminosae* family indicating host advantage and likely higher symbiotic performance (Oono et al., 2010). Terminal bacteroid differentiation is the best elucidated in the *S. meliloti* – *M. truncatula* symbiosis. In *M. truncatula* nodules, the most visible events of terminal bacteroid differentiation occur in zone II. Multiplication of bacteroids stops in the middle of zone II where cell elongation and uniform amplification of the multiple replicons by endoreduplication cycles begin. Along 2–3 cell layers at the border of zone II and III (called interzone) sudden growth of bacteroids is visible reaching practically their final size, however, nitrogen-fixation takes place only in zone III.

## HOST PEPTIDES GOVERN BACTEROID DIFFERENTIATION

**FIGURE 2:**
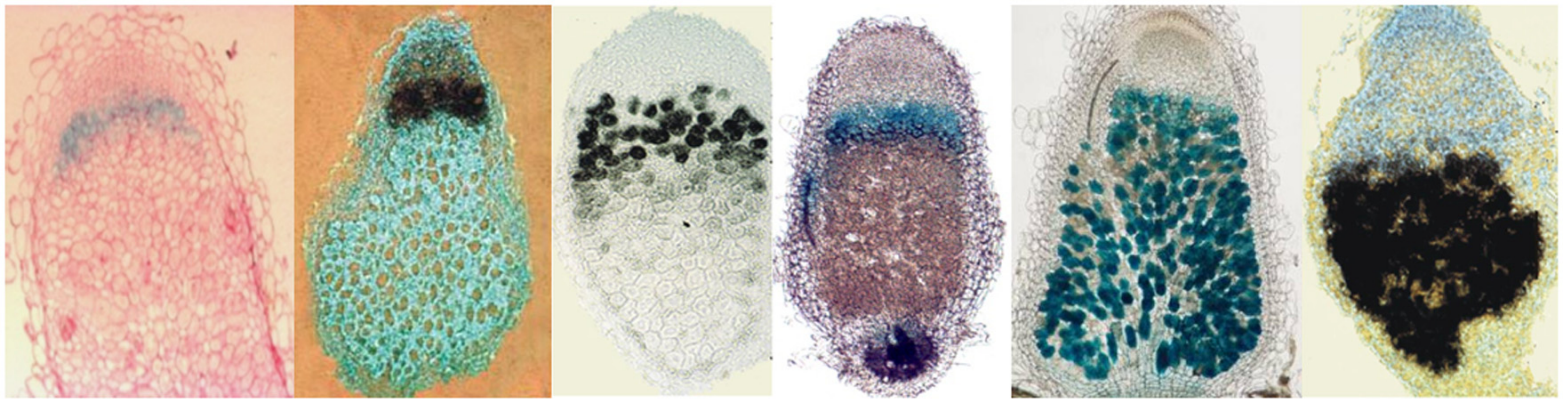
**Differential expression of *symPEP* genes in *M. truncatula* nodules.** Black signal: *in situ* hybridization, blue signal: GUS activity of *symPEP* promoter-GUS fusions in transgenic nodules.

Comparison of nodule transcriptomes of legumes with reversible and irreversible bacteroid differentiation revealed the existence of several hundreds of small genes that were only present in the genome of those host plants where bacteroid differentiation was terminal. In *M. truncatula* the nodule cells produce at least 600 nodule-specific symbiotic peptides (symPEPs). The *symPEP* genes are only activated in the *S. meliloti* infected polyploid symbiotic cells (Kevei et al., 2002; Mergaert et al., 2003), however certain sets at the earlier, others during the later stages of nodule development. A large portion, more than 500 genes encode nodule-specific cysteine-rich (NCR) peptides (Mergaert et al., 2003; Alunni et al., 2007; Nallu et al., 2014). The NCR peptides are targeted to the bacteroids and when their delivery to the endosymbionts was blocked, bacteroid differentiation was abolished demonstrating that the peptides are responsible for terminal differentiation of *S. meliloti* bacteroids (Van de Velde et al., 2010). The high sequence variety and the characteristic expression patterns of *NCR* genes suggest diversity in their functions, modes of action and bacterial targets at different stages of bacteroid maturation (**Figure 2**). However, why does the host cell produce an arsenal of NCRs? What can be the advantage of such a diverse peptide repertoire? Is it necessary for interaction of the host with various bacteria? The symbiotic partners of *M. truncatula* are *S. meliloti* and *S. medicae*, however in the soil there are countless strain variants of both species. *M. truncatula* is also represented by many different ecotypes and accessions differing in the number, sequences, and expression profile of *NCR* genes and in their symbiotic interactions with different *S. meliloti* and *S. medicae* strains (Nallu et al., 2014; Roux et al., 2014). While a nodule contains a single bacterium type, the different nodules on the same root system may possess distinct bacterial populations. It is possible that the plant recognizing the various endosymbionts manipulates them with a strain-specific repertoire of peptides. These differences can add an additional control level for host-symbiont specificity and thereby for nodulation efficiency.

Although symPEPs represent unique peptide classes, their structures resemble to antimicrobial peptides (AMPs). AMPs with broad spectrum of microbial cell-killing activity are most frequently cationic provoking cell death by pore formation, membrane disruption and consequent lysis of microbial cells. The fact that the cell division ability is definitively lost during endosymbiont differentiation indicates that at least certain symPEPs have antimicrobial activities. Treatment of bacteria with synthetic cationic NCRs indeed provoked rapid and efficient dose-dependent elimination of various Gram-negative and Gram-positive bacteria including important human and plant pathogens (Van de Velde et al., 2010; Tiricz et al., 2013). This ex-planta killing effect correlated with permeabilization of microbial membranes, however, symPEPs in their natural environment – in the nodule cells – do not permeabilize the bacterial membranes and do not kill the endosymbionts. Most likely the peptide concentrations in the nodules are significantly lower than those applied in the *in vitro* assays. Moreover cationic peptides are produced together with anionic and neutral peptides in the same cell, and possible combination of a few tens or hundreds of peptides with various charge and hydrophobicity might neutralize the direct bactericidal effect of the cationic peptides.

The involvement of AMPs or AMP-like peptides is not unique for *Rhizobium*-legume symbiosis. In the weevil* Sitophilus,* the symbiotic cells produce the antimicrobial peptide coleoptericin-A (ColA) which provokes the development of giant filamentous endosymbionts by inhibiting cell division and protects the neighboring insect tissues from bacterial invasion (Login et al., 2011). In this system a single peptide is sufficient for differentiation of the obligate vertically transmitted endosymbiont unlike nodules that operate with hundreds of symPEPs and can host innumerable strain variants as their endosymbionts. In the aphid-*Buchnera* symbiosis, the host cells also produce bacteriocyte-specific peptides including cysteine rich peptides (BCRs) which resemble the *Medicago* NCR peptides, however the functions of these symbiotic peptides have not been reported yet (Shigenobu and Stern, 2013).

## NCR247: AN EXAMPLE FOR MULTI-TARGET HOST EFFECTOR

Transcriptome analysis of *M. truncatula* nodules at different stages of their development, laser microdissection of nodule regions, in situ hybridization, immunolocalization of selected peptides, and symPEP promoter-reporter gene fusions in transgenic nodules allow mapping the action of individual peptides in the symbiotic cells from the early infection until the late nitrogen fixation state. NCR247 is expressed in the older cell layers of zone II and in the interzone where bacterial cell division stops and remarkable elongation of the endosymbionts occurs (Farkas et al., 2014). This small cationic peptide effectively killed various microbes *in vitro* and the *in silico* analysis indicated its extreme protein binding capacities. FITC-labeled NCR247 entered the bacterial cytosol where its interactions with numerous bacterial proteins were possible. Binding partners were identified by treatment of *S. meliloti* bacteria or bacteroids with StrepII/FLAG-tagged peptides followed by affinity chromatography and identification of interacting partners with LC-MS/MS and Western analysis (Farkas et al., 2014).

One of the interactors was the FtsZ cell division protein playing a crucial primary role in cell division. A number of antibiotic peptides are known to exert bactericidal or bacteriostatic effect through the interaction with FtsZ, inhibiting its polymerization thereby hindering proper Z-ring and septum formation (Handler et al., 2008). NCR247 was co-purified with FtsZ from the bacterial cytoplasm and was shown to disrupt septum formation. NCR035 exhibiting *in vitro* also bactericidal effect and produced in the same symbiotic cells as NCR247 accumulates at the division septum which indicates simultaneous or consecutive action of these peptides and evolution of multiple host strategies to inhibit endosymbiont proliferation. Another study showed that expression of important cell division genes, including genes required for Z-ring function, were strongly attenuated in cells treated by NCR247 (Penterman et al., 2014). Pretreatment of bacteria with sub-lethal NCR247 concentrations abolished localization of FITC-NCR035 to the septum and provoked cell elongation (Farkas et al., 2014).

Ribosomal proteins were the most abundant NCR247 interacting partners. NCR247 was observed to strongly inhibit bacterial protein synthesis in a dose-dependent manner both *in vivo* and *in vitro* (Farkas et al., 2014). These results suggested that one mode of the NCR247 peptide action is binding to the ribosomes both in bacterial cells and bacteroids. Interestingly, an altered pattern and reduced complexity of the interacting proteins were observed in the bacteroids. Accordingly the general expression level of ribosomal proteins was in average 20-fold lower in the bacteroids than in the free-living cells with different relative abundance of transcripts of individual ribosomal proteins. Ribosome diversification in bacteroids may have a significant role by contributing to the advanced translation of specific proteins thereby supporting the specialized, energy-demanding physiology of highly abundant nitrogen fixation function.

The GroEL chaperon was also a direct interacting partner of NCR247 (Farkas et al., 2014). Out of the 5 GroEL proteins, GroEL1 or GroEL2 is sufficient for survival while GroEL1 expressed at high level in the nodule is essential for symbiosis (Bittner et al., 2007). It is needed for full activation of the nodulation genes and assembly of the nitrogenase complex. GroEL possesses extreme functional versatility by interacting with hundreds of proteins. The NCR247-GroEL1 interaction can have consequences directly on GroEL but indirectly also on the GroEL substrates and the associated biological processes. Absence of GroEL1 severely affected bacterial infection and the maintenance and differentiation of bacteroids demonstrating a general need for GroEL1 in all stages of nitrogen fixing nodule development.

The involvement of GroEL and host peptides in microbe-host interactions is not unique for *Rhizobium*-legume symbiosis. In the weevil symbiotic cells coleoptericin-A (ColA) interacts also with GroEL (Login et al., 2011). GroEL also plays an important role in the maintenance of endosymbionts (Moran, 1996; Kupper et al., 2014). As most symbiotic systems are as yet unexplored and high-throughput genomic and proteomic tools are only recently available, we can only predict that host peptides-mediated endosymbiont differentiation, likewise genome amplification of host cells and terminally differentiated endosymbionts are general strategies of symbiosis.

## CONCLUSION

Symbiotic and pathogenic bacteria use similar approaches to interact with their hosts and to survive within host cells, even if the results of these interactions are strikingly different. Plants and animals can generate innate immune responses to microorganisms upon the perception of MAMPs (microorganism-associated molecular patterns). This perception results in the activation of signaling cascades, and the production of antimicrobial effectors. AMP-like host peptides such as the *M. truncatula* NCR peptides or the weevil ColA antimicrobial peptide play pivotal and multi-faceted roles in controlling the multiplication and differentiation of endosymbionts, thereby restricting the presence of bacteria to the symbiotic cells. Thus, host organisms utilize these effector peptides to tame and even hire selected microbial invaders for service.

## Conflict of Interest Statement

The authors declare that the research was conducted in the absence of any commercial or financial relationships that could be construed as a potential conflict of interest.
